# Elucidating the context for implementing nonpharmacologic care for neonatal opioid withdrawal syndrome: a qualitative study of perinatal nurses

**DOI:** 10.1186/s12887-021-02955-y

**Published:** 2021-11-04

**Authors:** Clayton J. Shuman, Roxanne Wilson, Katherine VanAntwerp, Mikayla Morgan, Ashley Weber

**Affiliations:** 1 School of Nursing, University of Michigan, 400 N. Ingalls, Ste. 4162, Ann Arbor, MI USA; 2 Institute for Healthcare Policy and Innovation, University of Michigan, Ann Arbor, MI USA; 3 Center for the Study of Drugs, Alcohol, Smoking, and Health, University of Michigan, Ann Arbor, MI USA; 4 Department of Nursing, St. Cloud State University, St. Cloud, MN USA; 5 St. Cloud Hospital, St. Cloud, MN USA; 6 College of Nursing, University of Cincinnati, Cincinnati, OH USA

**Keywords:** Neonatal abstinence syndrome, Perinatal substance use, Maternal engagement, Nonpharmacologic care, Neonatal opioid withdrawal syndrome, Implementation

## Abstract

**Background:**

Up to 95% of neonates exposed to opioids in utero experience neonatal opioid withdrawal syndrome at birth. Nonpharmacologic approaches (e.g., breastfeeding; rooming-in; skin-to-skin care) are evidence-based and should be implemented. These approaches, especially breastfeeding, rely on engagement of the neonates’ mothers to help deliver them. However, little is known about the structural and social dynamic context barriers and facilitators to implementing maternal-delivered nonpharmacologic care.

**Methods:**

Using a qualitative descriptive design, perinatal nurses from a Midwest United States hospital family birthing center, neonatal intensive care unit, and inpatient pediatric unit were interviewed. These units were involved in caring for mothers and neonates affected by opioid use. Telephone interviews followed a semi-structured interview guide developed for this study, were audio-recorded, and lasted about 30–60 min. Interviews were transcribed verbatim and independently analyzed by five investigators using the constant comparative method. Themes were discussed until reaching consensus and subsequently mapped to a conceptual model adapted for this study.

**Results:**

Twenty-one nurses participated in this study (family birth center, *n* = 9; neonatal intensive care, *n* = 6; pediatrics, *n* = 6). Analysis resulted in four major themes: 1) Lack of education and resources provided to staff and mothers; 2) Importance of interdisciplinary and intradisciplinary care coordination; 3) Flexibility in nurse staffing models for neonatal opioid withdrawal syndrome; and 4) Unit architecture and layout affects maternal involvement. Minor themes supported each of the four major themes. All themes mapped to the conceptual model.

**Conclusions:**

This study provides a more comprehensive understanding of the barriers and facilitators affecting implementation of maternal involvement in nonpharmacologic care of newborns with neonatal opioid withdrawal syndrome. Future efforts implementing nonpharmacologic approaches must consider the context factors affecting implementation, including structural and social factors within the units, hospital, and broader community.

**Supplementary Information:**

The online version contains supplementary material available at 10.1186/s12887-021-02955-y.

## Background

Opioid use and misuse in the United States continues to affect neonates [1], with one neonate born suffering from opioid withdrawal every 15 min [2]. Up to 95% of neonates exposed to opioids in utero experience neonatal opioid withdrawal syndrome (NOWS) at birth [3]. NOWS begins shortly after birth and results from termination of the mother’s opioid supply after the umbilical cord is severed [4]. NOWS symptoms vary in clinical presentation, ranging from minor (e.g., mild tremors, irritability) to more severe (e.g., gastrointestinal distress, seizures) [5]. Common outcomes for neonates diagnosed with NOWS include admission to a neonatal intensive care unit (NICU), receipt of pharmacological treatment (e.g., morphine), prolonged hospitalizations, increased costs and resource utilization, and concurrent birth complications (e.g., jaundice, low birth weight, feeding difficulties) [3]. In addition to adverse clinical outcomes, neonates with NOWS are more likely to be separated from their mothers during their hospital stay and after discharge which can affect maternal-infant bonding, family dynamics, and long-term child health and safety [3–5].

Treatment for NOWS is informed by routine assessment of symptom severity (e.g., Finnegan scoring) and often involves both evidence-based nonpharmacologic as first line treatment (e.g., breastfeeding, skin-to-skin care) and pharmacologic approaches [6]. The Eat, Sleep, Console approach has garnered attention nationally and prioritizes nonpharmacologic interventions before initiating pharmacologic interventions [7]. Nonpharmacological interventions have been shown to decrease infant lengths of stay and need for pharmacologic interventions [8]. However, to achieve these outcomes, these evidence-based practices for NOWS must be implemented in clinical practice [6].

Nonpharmacologic approaches for NOWS treatment include a constellation of interventions such as soothing techniques (e.g., swaddling, pacifier use, low stimulation environment) and nutrition and feeding approaches (e.g., breastfeeding, smaller and more frequent feedings, use of infant formulas formulated for sensitive gastrointestinal systems) [8–13]. Optimal treatment of NOWS includes delivery of rooming-in [8–11], breastfeeding [12, 13], and skin-to-skin contact [12, 13]; thus the mother must be considered as a primary provider of nonpharmacologic treatment. However, mothers are often not present at the bedsides of neonate’s with NOWS, leading to low breastfeeding rates and limited delivery of skin-to-skin care for this population [4, 9, 10, 12, 14, 15]. Failure to implement these approaches are due in part to individual-level barriers such as clinician attitudes and stigma, maternal feelings of guilt, and lack of clinician education on NOWS [15–17].

In additional to individual-level factors, the clinical context may also act as a barrier or facilitator to implementation. However, little is known about how the clinical context (e.g., health system, perinatal unit) affects engagement of mothers in implementation of nonpharmacologic care. Families affected by NOWS have recognized contextual barriers to maternal engagement in care, such as inconsistencies among clinicians and repeated transfers between units (e.g., labor and delivery, NICU, pediatrics) [18]. However, a more comprehensive understanding of the contextual barriers and facilitators to implementing maternal-delivered nonpharmacological care is needed to help hospitals, perinatal units, and community service organizations improve neonatal and maternal outcomes related to NOWS. The purpose of this qualitative exploratory study was to examine perinatal, neonatal, and pediatric nurse’s perceptions regarding the contextual barriers and facilitators related to maternal engagement in and delivery of nonpharmacologic care of neonates with NOWS.

## Methods

### Design

A qualitative descriptive design was used to identify nurse-perceived contextual barriers and facilitators. A semi-structured interview guide was developed and implemented to elicit the perceptions of perinatal, pediatric, and neonatal intensive care nurses caring for opioid-exposed mothers and neonates (see below). The study was approved by the University of Michigan institutional review board and approved by the study site’s institutional review board.

### Conceptual model

**Fig. 1:**
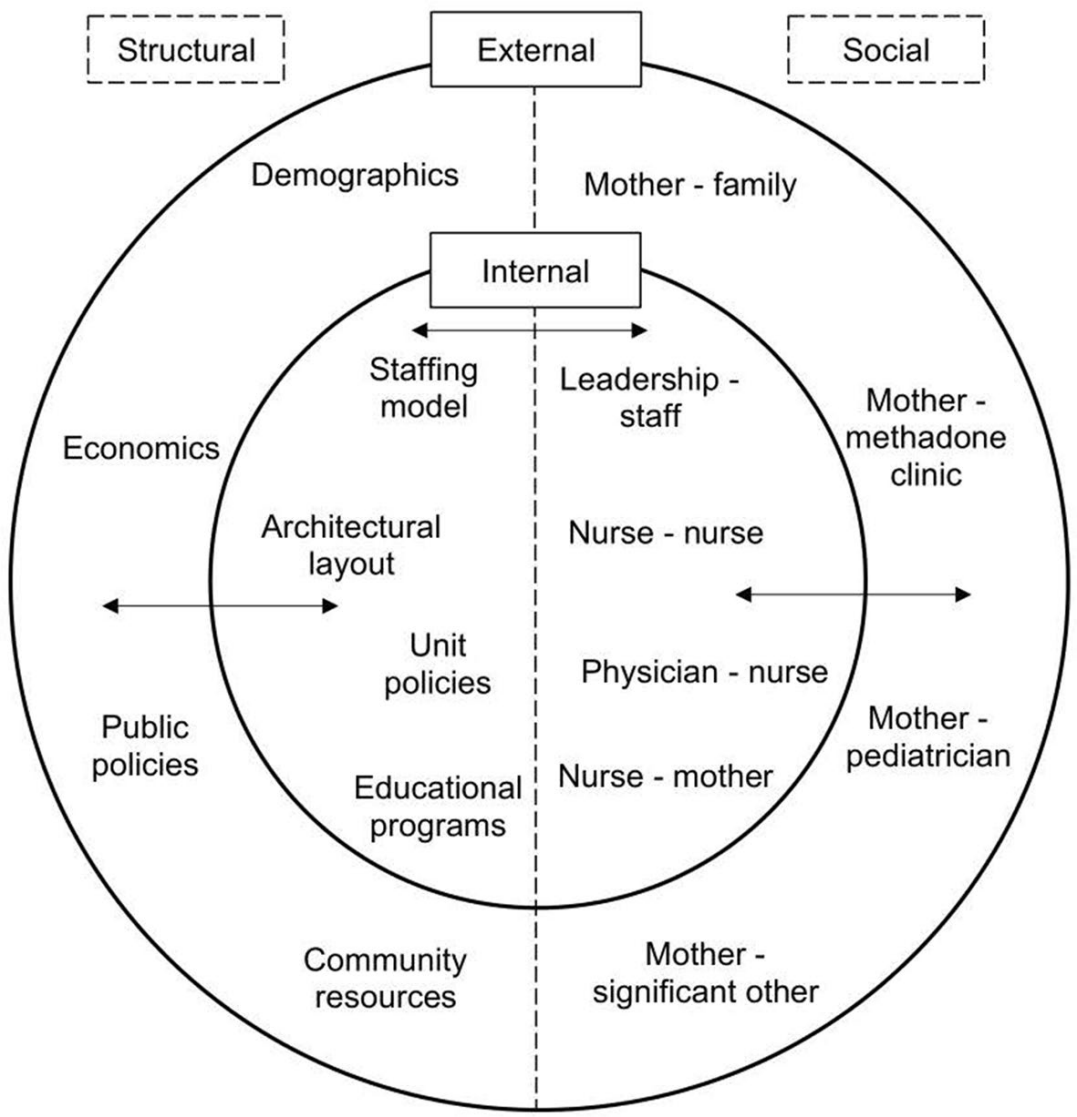
External and internal social and structural dynamic factors. Legend: Inner circle = internal to the hospital; Outer circle = external to the hospital; Left side of the model = structural factors; Right side of the model = social dynamic factors; Double-sided arrows = interactions between structural and social dynamic factors or across internal and external settings. Dash = relationship between two stakeholders

This study was informed by an adapted conceptual model originally developed by Shuman and colleagues [19]. The clinical context was conceptualized as comprising internal (within the hospital) and external (in the community or society) structural and social dynamic factors (Fig. 1). Internal structural factors are relatively static and include elements such as clinical staffing models, architectural layout and features, unit policies, and educational programs. External structural factors may include the demographics and economics of the surrounding community, public policies, and community resources. Internal social dynamic factors refer to the roles and relationships of individuals and groups within the clinical context, such as the relationships among leadership and staff, physicians and nurses, and nurses and mothers. Social dynamic factors external to the hospital may include relationships among mothers and their families (e.g., significant other, parents), mothers and their public health departments or community providers (e.g., methadone clinics, primary care providers, pediatricians).

### Setting and participants

Data were collected in 2018 from nurses at a regional hospital in Minnesota with over 70 neonates diagnosed with NOWS between July 2016 and June 2018. All units caring for opioid-exposed neonates and mothers were included: family birth center (labor and delivery, postpartum and nursery), neonatal intensive care unit (NICU), and inpatient general pediatric unit. Neonates not requiring pharmacologic treatment for NOWS and considered otherwise stable remained at the mothers’ bedsides in the family birth center and received care from postpartum nurses trained in NOWS assessment and treatment. Virtual special care was provided to neonates if mothers were eligible for discharge, but their neonates remained on the unit. Virtual special care was not a physical unit and involved monitoring, assessment, and nonpharmacologic treatment while the neonate physically remained in the FBC. Neonates requiring pharmacologic treatment for NOWS were admitted to the pediatric unit. If neonates presented with additional complicating factors (e.g., prematurity), they were admitted to the Level 3 NICU consisting of 30 single patient rooms.

Participants were recruited from these study units who met the following inclusion criteria: 1) ≥18 years of age; 2) licensed as a registered nurse; 3) employed a minimum of 12 h per week; 4) designated as staff on a study unit (not float pool or agency); 5) have cared for neonates diagnosed with NOWS in the past 3 years at the study site; and 6) able to speak and understand English.

### Procedures

Eligible participants received an email invitation developed by the research team and sent by the nursing director of each study unit. Those willing to participate in the study enrolled via a web-based form. One member of the study team contacted each participant via telephone to schedule an interview date and time. All interviews were conducted between July and September 2018. Prior to each interview, a study team member reviewed informed consent, answered questions, and obtained verbal consent. A research assistant (KP) and one co-investigator (RW) conducted the interviews independently by telephone. Telephone interviews for qualitative descriptive studies have been shown to be as effective as face-to-face interviews, especially when using semi-structured interview guides [20–23]. Interviews lasted approximately 30–60 min and were audio-recorded. Participants were offered a $20 cash card following the interview.

### Instrument

A semi-structured interview guide was developed and implemented to facilitate interviews and data collection. Topics in the guide were informed by relevant extant literature and external and internal contextual constructs identified in the Shuman and colleagues conceptual model [19] adapted for this study and those included in the Consolidated Framework for Implementation Research (CFIR) model [24]. The guide included introductory and closing scripts, open-ended questions, and probes (see Supplemental Content). Questions and probes were designed to elicit and describe nurse perceptions of contextual factors affecting engagement of mothers in the care of substance-exposed neonates. Although the interview guide was not specific to opioids, all participants primarily focused on opioid withdrawal. This was not surprising because opioid-exposure is most often associated with neonatal withdrawal symptoms [3]. Additional questions in the guide were used to describe individual level (e.g., nurse, mother, neonate) barriers and facilitators, which have been described previously [24]. The guide was rigorously reviewed by the investigative team and stakeholders at the study site (e.g., unit nursing directors, clinical nurse specialists, medical directors) for relevance and comprehensiveness. The flow and content of the guide was tested with two nursing students with clinical and research experience in maternal-infant health.

### Data analysis

Interviews were transcribed verbatim and reviewed for transcription accuracy by comparing transcribed files with audio files. Using the constant comparative methods of Glaser and Strauss [25, 26], all members of the investigative team individually performed initial coding (e.g., minor themes); the team then compared and discussed codes until reaching agreement. Investigators independently organized minor themes into major themes; themes were then compared and discussed until consensus was reached. Qualitative rigor was demonstrated by establishing trustworthiness [25] through assuring credibility (peer debriefing, member-checking, and prolonged engagement), dependability (inquiry audit), and confirmability (reflexivity) [25, 26]. After identifying themes, investigators mapped them to the conceptual model and discussed them until achieving unanimity.

## Results

### Participant demographics

A total of 34 nurse participants who met inclusion criteria enrolled in this study (Table 1). Thirteen of the enrollees were unable to be reached via telephone or email or were unable to schedule an interview date and time. Thus, 21 nurses participated in individual, semi-structured interviews. Nine nurses were from the family birth center, six were from the NICU, and six were from the pediatric unit. Participants were mostly female (95%), Caucasian (100%), and had a mean age of 37 years (SD = 10).

**Table 1:** Participant Demographics (*N* = 21)

	N (%)
**Sex**	
Female	20 (95%)
**Highest Nurse Educational Level**	
Associates	1 (5%)
Bachelors	18 (86%)
Masters	2 (9%)
**Advanced Practice Nurse**	
Yes	1 (5%)
No	20 (95%)
**Years of Experience as a Registered Nurse**	
0–2 years	4 (19%)
3–5 years	4 (19%)
6–10 years	4 (19%)
> 10 years	9 (43%)
**Years Employed as a Registered Nurse at the Study Hospital**	
0–2 years	5 (24%)
3–5 years	5 (24%)
6–10 years	3 (14%)
> 10 years	8 (38%)
**Years Employed as a Registered Nurse by their Current Unit**	
0–2 years	5 (25%)
3–5 years	4 (19%)
6–10 years	5 (24%)
> 10 years	7 (33%)

### Themes

**Fig. 2:**
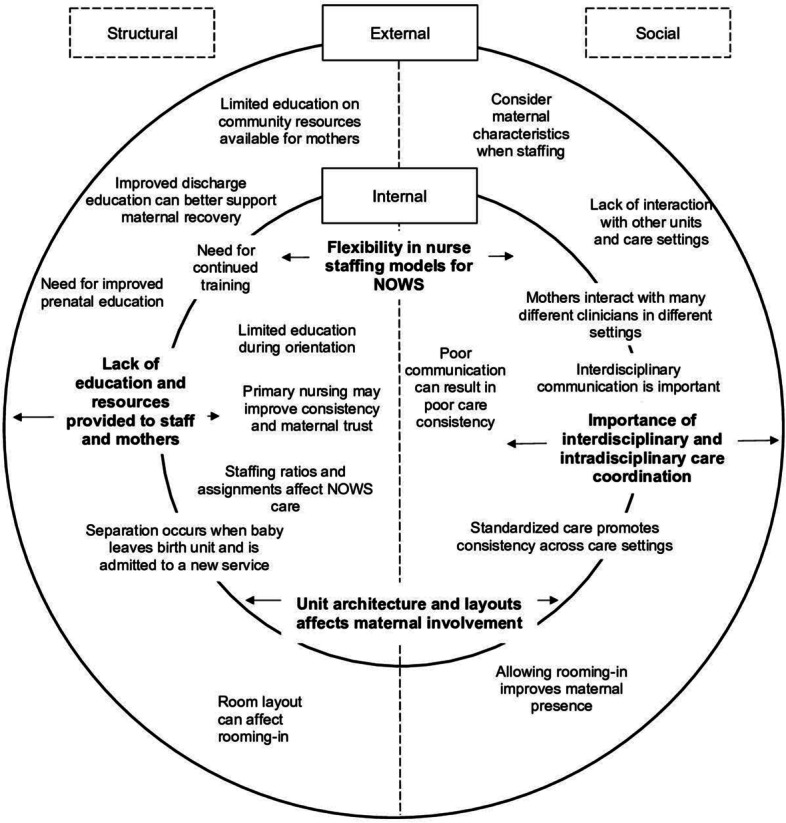
Themes mapped to the conceptual model. Legend: Bold font = major theme; Normal font = minor theme; Left side of model = structural factors; Right side of model = social dynamic factors; Inner circle = factors internal to the hospital; Outer circle = factors external to the hospital. Double-sided arrows = theme includes structural and social factors or spans internal and external settings

Four major themes were identified: 1) Lack of education and resources provided to staff and mothers; 2) Importance of interdisciplinary and intradisciplinary care coordination; 3) Flexibility in nurse staffing models for NOWS; and 4) Unit architecture and layout affects maternal involvement. Minor themes supported each of the four major themes (Table 2). To demonstrate application of these themes to future research, clinical practice, and public policy, each theme was mapped to our conceptual model (Fig. 2).

**Table 2:** Themes and exemplar quotes

Major Theme	Minor theme	Exemplar quotes
**Lack of education and resources provided to staff and mothers**	Limited education during orientation	When I was first hired in the NICU, we were shown an assessment video to show how they assess early withdrawal babies; but that’s all. [When] I train new staff, I am trying to help make sure that they don’t form biases ahead of time...and trying to teach the newer, younger staff.
	Need for continued training	I just think we [perinatal nurses] need the whole opioid addiction education. We just don’t get any of it really. I think just by continuing to educate nurses as well to help give them skills to pass along to their patients would help as well, because I feel like some of the knowledge is limited and some of it’s just experience-based.
	Limited education on community resources available for mothers	[Nurses should] be more aware of the resources in the area and probably just like more education for us to teach the parents. Nurses should be more educated in the aspect of social work though because let’s say we’re on the weekend and we can’t necessarily get ahold of a social worker.
	Improved discharge education can better support maternal recovery	More information in the community [would be helpful at discharge for these mothers]...but as far as mom goes, as far as helping her to stop using substances, I think that—I wish we had a little bit more resources for that. I don’t feel like enough is really done to help them [mother’s] at discharge to tell ya the truth.
	Need for improved prenatal education	I guess the scenario that could use a little more improvement is a little more prenatal preparation and education. We need to do some more education with these moms when they’re still pregnant.
**Importance of interdisciplinary and intradisciplinary care coordination**	Standardized care promotes consistency across care settings	[We promote consistency by] reading the care plan, following the care plan, [in order to] stay consistent with what our - our end goal is.
	Poor communication can result in poor care consistency	Consistency between units is based solely on the quality of the report that you’re being given...they’re not familiar with each other’s policies, and things like that, so, um, they don’t always know what they need—they don’t always know what the other nurse needs to know. I can think of not too long ago, um, the baby was receiving routine scores of 13 to 15 [in Family Birthing], which are really high. And the baby got over there [to NICU] and once we scored, it was actually 6 to 8. All we knew were the scores, not why they scored higher.
	Interdisciplinary communication is important	I feel like a lot of times there is, um, kind of like this process of the mothers being a little bit like manipulative of them [nurses] kind of trying to play “oh, but the doctor didn’t say this,” or, and I think sometimes the barrier of us not being completely unified and having like a one, one time to go in and discuss it with everybody involved at that point...I feel like the barrier is just not having essentially like a care conference with the patient with the providers and the nursing staff and with a social worker, doctors, whatever. I think that a lot of times the problem with the congruency isn’t congruency of nurses to nurses. It’s more like well the doctor said this, but that’s so-and-so doctor, and now this one’s on call, and the social worker, but she’s not here on the weekend...a lot of times where the problem lies is within different disciplines.
**Flexibility in nurse staffing models for NOWS**	Primary nursing may improve consistency and maternal trust	If the same nurse has been here on Friday, she’s gonna be here all weekend then keep giving that child back to that nurse, cuz she gets to know that mom and that baby really well. [I recommend] one nurse for the day and one nurse for the night to keep consistency throughout that [patient’s stay].
	Consider maternal characteristics when staffing	In the NAS [neonatal abstinence syndrome] situation, you’re - you’re doing more education on those kind of primary situations. [A barrier is] what acuity we put mom at. You know, babies who are NAS scoring are up, you know, at an acuity level automatically, but that doesn’t mean that, um, we factor in mom’s view and mom’s educational needs.
	Mothers interact with many different clinicians in different settings	Having so many different nurses for each [patient]...it would be inconsistency I guess with nurses. Um, where I feel like if we did have the same nurses who were working, you know, 3 days in a row, three twelves in a row, if we could staff them with these - with these couplets, I feel like that would be pretty consistent. You might labor a patient and then send them to a postpartum nurse...or you might have had someone else labor them and get them sent to you. Some people only work one or the other area in our unit, and if the baby’s not well, it might go to NICU and stay there.
	Staffing ratios and assignments affect NOWS care	These babies often would benefit from lower staffing ratios. Just assignments, in general, can be a problem. Sometimes you don’t have a choice, like I said, how—what an NAS baby is put—what kind of assignment they’re put into.
**Unit architecture and layout affects maternal involvement**	Allowing rooming-in improves maternal presence	One of the reasons why, um, we get the NAS babies transferred over to the pediatric unit is the moms are able to stay with them over there [pediatric unit]. I think the fact that we [pediatric unit] have, um, private rooms is huge because if the mothers are allowed to, um, they can be there, and we can work with them there for 24 h a day.
	Separation occurs when baby leaves birth unit and is admitted to a new service	There’s no separation [in the Family Birth Center]. The separation would come if baby goes to NICU [and mom stays in Family Birth Center]. if baby needs that special care [virtual special care nursery], they’re able to be with their moms instead of going to NICU…which I think before that, usually it was kind of taking baby out of their rooms at that time and monitoring babies with the mom not even being involved.
	Room layout can affect rooming-in	A parent can stay at the bedside, um, but there is not their own bathroom and - and such [in the NICU]. Whereas now, we - we, um, house them [NOWS neonates] more on our pediatric unit...it’s a little bigger rooms, and they have bathrooms in their rooms. We don’t give any food on the unit [pediatric], and I know a lot of parents complain about that, um, that we don’t provide them any food or snacks.

#### Lack of education and resources provided to staff and mothers

Nurses described a lack of comprehensive unit- or organization-provided NOWS and opioid use disorder (OUD) education to effectively care for this patient population. Several nurses recalled NOWS education received during new-hire orientation, but noted it was exclusive to NOWS scoring assessment skills (e.g., Finnegan scoring) without attention to maternal OUD and evidence-based NOWS treatment approaches. Experienced nurses feared new hires did not receive adequate education on how to care for neonates with NOWS, especially concerning the trajectory of maternal OUD and treatment, and the numerous, interacting complex social and family dynamics (e.g., marital status; family proximity; societal stigma). Since hospital provided education was considered lacking, nurses relied on various internet resources (e.g., Google) to learn more about how to care for families affected by NOWS but noted internet resources could be unreliable. Many nurses vocalized experience working with families affected by NOWS; however, they felt their previous experiences were not sufficient to prepare them to care for these maternal-infant dyads. They stated more formalized education from the hospital surrounding maternal OUD (e.g., pathophysiology of addiction; psychology of addiction; trajectory of recovery), NOWS (e.g., long-term infant health and social outcomes; evidence-based treatment approaches), and engaging mothers in treatment of NOWS (e.g., therapeutic communication with mothers with OUD; understanding adverse childhood experiences and trauma) would supplement their experiences and increase their ability to provide quality care to neonates and families affected by NOWS. One nurse summarized, “I just think we [perinatal nurses] need the whole opioid addiction education…we just don’t get any of it really.”

In addition to nursing education, participants stressed the need for hospitals and communities to enhance the education mothers receive regarding NOWS. A lack in maternal knowledge about NOWS was thought to negatively affect a mother’s willingness to engage in neonatal care and implement nonpharmacologic interventions. Several nurses proposed increasing prenatal education about NOWS in community and primary care settings to better prepare mothers for what to expect after delivery. This education could be delivered during prenatal care visits, addiction treatment clinic visits, or through community outreach initiatives. However, nurses acknowledged that many mothers do not receive prenatal care and suggested education provided by labor nurses could help set the stage for the entire admission. At discharge, nurses felt mothers were not adequately prepared for a successful transition to home. Specifically, nurses reported mothers were not provided adequate opioid cessation resources at discharge and/or unit and organizational collaboration with community resources and treatment centers were unknown or not well-articulated. Nurses wanted to help mothers and neonates with transition from the hospital to home, but they did not fully understand the trajectory of these patients during and following transitions back to the community.

Nurses in the NICU and pediatric unit emphasized a lack unit and hospital-provided nursing education about challenges faced by mothers with OUD and what resources may be available to help them (e.g., transportation, housing, home visits). Since mothers are not admitted to pediatric or neonatal units, some of these nurses considered the family as “the patient” but felt a strong sense of protection for the neonate who is vulnerable as the “primary patient”. In addition, they felt a sense of vulnerability in trying to engage the mother while documenting her actions. This was particularly challenging because nurses understood their charting and interactions could impact findings of Child Protection Services (CPS), yet they had little-to-no interaction with CPS to explain or discuss their concerns.

Many nurses relied on social workers and case managers to connect mothers with community resources (e.g., breastfeeding support, local and state resources, case management, residential treatment, childcare). However, nurses working night or weekend shifts felt less knowledgeable about the availability of hospital and community resources because social workers and case managers were less accessible. Nurses recommended increasing hospital-provided education and collaboration with social work and case management across shifts and in the continuum of care. This would involve considering innovative solutions to utilize social work and case management staff and resources on nights and weekends, when most mothers visited the NICU or pediatric units or when some were discharged.

Nurses identified increased hospital-provided education on addiction, recovery, and post discharge support is needed to improve their practice in interacting with the parents during the neonate’s stay and in planning for transition to the community and post discharge care. Without hospital-provided education, nurses described feeling incompetent and unconfident in communicating with mothers with OUD. They felt additional on-the-job training and education could significantly improve their understanding of the mothers’ addiction and recovery experiences, leading to increased confidence in engaging these mothers in neonatal care. On the conceptual model, this theme mapped to structural context factors both internal (e.g., hospital-provided education) and external (e.g., community resources for pregnant women with OUD) to the hospital.

#### Importance of interdisciplinary and intradisciplinary care coordination

Care coordination was identified as a significant factor contributing to maternal engagement in implementation of nonpharmacologic care and relied on communication across settings (e.g., prenatal, hospital, home/community), interdisciplinary communication, or communication between different providers (e.g., nurses, physicians, social workers), and intradisciplinary communication, or communication within one set of providers (e.g., nurses). Interdisciplinary communication, as described by the nurses from all types of shifts, was compromised between members of the care team (e.g., physician, social work, case manager, lactation consultant, nurse). For example, one nurse stated, “I think that a lot of times the problem with the congruency isn’t congruency of nurses to nurses. It’s more like well the doctor said this, but that’s so-and-so doctor, and now this one’s on call, and the social worker, but she’s not here on the weekend...a lot of times where the problem lies is within different disciplines.”

Nurses explained a lack of opportunity to comprehensively discuss care among disciplines contributed to inconsistent care coordination and transition planning (e.g., labor unit to NICU; discharge). One nurse said, “I don’t know what social work does once they’re out of our hands.” Although interdisciplinary care rounds facilitated discussion across disciplines, nurses accentuated a lack of communication regarding NOWS treatment which ultimately led to incongruencies in care and poorer outcomes. Further, mothers were often not involved in planning: “I think that there’s just a lot of discussion not in front of the patient [mother] as that transition [to community] and to make that work and when it’s appropriate and when it’s not.”

While describing intradisciplinary communication and care coordination, nurses cited handoff report, nursing notes, bedside report, electronic medical record charting, and nursing care plans as communication modalities contributing to congruent patient care. However, they elaborated and stated the quality of handoff varies considerably across units and between nurses. The variation in the quality of handoff report or communication between units and nurses was described as a leading cause of decreased care quality and continuity. This was thought to adversely affect implementation of maternally provided nonpharmacologic care. For example, nurses suggested handoff reports should include discussion of effective and ineffective strategies used by the previous nurse to engage the mother.

Lack of interaction or connectedness between nurses from different units (e.g., labor and NICU) and care settings (e.g., hospital to community), was considered a critical barrier to care coordination, especially to continuation of breastfeeding and skin-to-skin care initiated at delivery. For example, breastfeeding support in a postpartum unit may not have been continued in the NICU when mothers were visiting neonates. In addition to inconsistencies in maternal engagement, nurses reported significant variation in how each unit scored neonates exhibiting symptoms of NOWS. As one nurse explained, labor nurses often scored neonates with suspected NOWS higher than the NICU nurses. Thus, nurses suggested implementing NOWS care practices across all units simultaneously to improve continuity and include community partners in interdisciplinary discharge planning. Standardized care across units and coordination across settings were described as effective strategies to improve engagement of mothers in neonatal care. These strategies were thought to improve maternal engagement by decreasing the level of care disruption mothers and neonates experience as they transition across shifts, units, and hospital and community settings. On the conceptual model, the theme of interdisciplinary and intradisciplinary care coordination mapped to social dynamic context factors, including communication among team members within the hospital (internal) and outside the hospital in the community (external).

#### Flexibility in nurse staffing models for NOWS

Nurses described staffing as a major unit and organizational factor contributing to the overall care of neonates with NOWS. Nurses, especially those practicing in the NICU, emphasized primary care nursing, a nursing model that assigns one nurse as a patient’s primary care provider for the duration of their hospitalization. Nurses noted primary care nursing, specifically with infants affected by NOWS, can improve care continuity and outcomes by affording increased opportunities for nurses to develop relationships the neonate’s family.

Similarly, FBC nurses also emphasized the importance of nursing consistency considering that a mother might labor with one nurse and postpartum with another. Nurses advocated for a more comprehensive approach to NOWS care – an approach that includes all hospital units (e.g., FBC, pediatrics, NICU) and community partners (e.g., treatment clinics, pediatricians, public health nurses) involved in the care of substance-exposed maternal-infant dyads. Episodic care, or unit-based care, can be overly distinct and specific to each unit. This could interfere with continuity of care, especially as nurses prepare mothers and neonates for transition from hospital into the community.

Nurses described neonates with NOWS and their families as having increased needs, both physical and educational, and these needs are often not prominent in determining nurse-patient assignments. As one participant stated, “If they [nurse] have a really heavy assignment and they have a baby who’s needing extra care or a mom who needs extra education regarding NAS [neonatal abstinence syndrome] scoring and they’re not able to be in there doing that…that is a barrier.” Because families affected by NOWS require increased education and social work resources and referrals, nurses felt these patients were of much higher acuity, not accounted for in current acuity indices or patient assignments. When discussing acuity levels and staffing, one nurse said, “[we don’t] factor in mom’s view and mom’s education needs.” Accounting for the heightened psychosocial and educational needs of mothers with OUD while making staffing decisions was thought to provide more time for nurses to effectively educate and engage mothers in neonatal care. This theme mapped to internal structural (staffing models) and social dynamic (nurse-mother relationship) context factors in the conceptual model.

#### Unit architecture and layout affects maternal involvement

The nurses felt the structural layout of the unit may facilitate or hinder maternal visitation and engagement. Many nurses described the ability for mothers to room-in with their neonate as a facilitator towards maternal involvement. Nurses recognized rooming-in contributed to increased maternal engagement in implementation of nonpharmacologic neonatal care such as breastfeeding and skin-to-skin care and provided numerous opportunities to educate the mother and connect her with community resources. Specifically, nurses described 24-h visitation policies and private rooms as two synergistic factors increasing a mother’s intention and ability to room-in with her neonate. However, other factors led to mothers having to leave the room including to go to the restroom, acquiring food, attending individual or group treatment sessions, obtaining methadone. Thus, nurses suggested utilizing rooms with bathrooms, delivering in-room meals for mothers, and providing OUD treatment, including methadone, using in-hospital resources until neonatal discharge.

Nurses from the labor and postpartum units emphasized the importance of the special care nursery in increasing maternal involvement. Nurses described a special care nursery as a virtual unit (not physical unit) which admits neonates with minor NOWS symptoms for monitoring. In the special care nursery, even after maternal discharge the mother had access to a full size bed and private bathroom in the neonate’s room. Nurses felt this model allowed for increased maternal engagement in neonatal care because the mother and neonate could remain in the same room while the mother recovers from delivery. Further, if required, the neonate would remain in the FBC as a “virtual special care” admission after the mother’s discharge. In addition, nurses described the pediatric unit as a prime location for neonatal treatment and they “encouraged moms to go with their babies to pediatrics” if the mother was discharged. Nurses felt that engagement of mothers in neonatal care was impacted by the type of unit where neonates received NOWS treatment, including the ability to room-in. On the conceptual model, this theme mapped to internal structural (e.g., unit architecture) and social dynamic (e.g., improved maternal presence at neonates’ bedsides) context factors.

## Discussion

Using a sample of perinatal, pediatric, and neonatal nurses, this study ascertained important contextual factors to consider when implementing nonpharmacologic care for NOWS, both within an acute care setting and in the broader community. The results of this study are timely because NOWS is a pressing public health crisis in the United States [1–3].

These findings compliment growing literature on the perceptions of clinicians and families affected by NOWS [4, 8, 10, 13, 27]. Previous studies identified individual-level barriers and facilitators to providing quality care and treatment for NOWS, as related to the clinician (e.g., nurse) or the mother. A common theme across many qualitative studies is the lack of clinician education regarding perinatal opioid use and NOWS. Results of this study support previous findings that perinatal units and healthcare organizations should identify and provide appropriate NOWS training and resources to nurses as well as families. Failure to provide education may result in 1) untrained clinical staff and ill-prepared mothers and 2) significant variation in clinician and maternal education resulting in poorer outcomes and inconsistent care.

Education of staff, although critical, is not sufficient to improve implementation and sustainability of evidence-based practices [28, 29]. Resources, care coordination, and care practices (e.g., staffing models) are also needed to support implementation. For example, Shuman and colleagues (2020) found that limited hospital- or community-based resources (e.g., lack transportation, financial assistance, childcare) often resulted in socioeconomic barriers that prevented mothers from being physically present at the bedside [15]. In the current study, resources promoting consistent care delivery practices and communication across perinatal units within a hospital were found to be essential to promoting breastfeeding, skin-to-skin care, and rooming-in among maternal-infant dyads affected by perinatal opioid use. It is important clinicians understand trajectories of these dyads, including clinical care and resources provided within the hospital, as well as outside the hospital in community-based settings. As argued by Spehr and colleagues, primary care providers are responsible for continuing to support maternally-provided care after discharge from the hospital [27]. Therefore, communication and knowledge regarding perinatal OUD and NOWS are important for hospital-based clinicians as well as community-based clinicians.

Families affected by NOWS often require more support and time from nurses [7]. Managing withdrawal symptoms involves constant monitoring and provision of nonpharmacologic (e.g., holding, pacifier) and sometimes requires pharmacologic interventions [4, 6]. Nurses with heavier assignments may not have time to provide this care or may not be able to fully care for other patients in their assignment effectively. Improving maternal involvement in neonatal care may reduce some of this workload for bedside nurses and improve patient care. However, it requires nurses to spend significant time training and preparing families to manage NOWS symptoms confidently. This attention, especially during the first few days following birth or diagnosis of NOWS, is critically important to effectively engaging these mothers in neonatal care which may ultimately lead to improved outcomes.

Notably, nurses described the physical layout and architecture of their units as important to engagement of mothers with OUD in the care of their neonates, especially private rooms with private bathrooms. Previous studies have demonstrated that neonates who room-in with their mothers have better outcomes [8–10]. However, for neonates cared for in NICUs, rooming-in may be less frequent depending on unit layout. Generally, the physical layout of NICUs in the United States are either a pod layout (e.g., many infant beds in a room) or offer single family private rooms (e.g., only one infant per room) [30–32]. Pod-style NICUs do not provide private space for mothers to deliver nonpharmacologic care such as breastfeeding or skin-to-skin [31–33]. Further, pods often do not offer a welcoming or conducive environment for 24/7 visitation [22–34]. Private room NICUs are costly and may not be a realistic solution for many hospitals [30, 31]. Even NICUs utilizing single room layouts can challenge rooming-in. For example, in this study, the NICU was structured as single family rooms with only neonatal beds and a couch for visitors, but no private bathrooms or adult beds. Consequently, more efforts may be warranted to identify other units (e.g., pediatric unit) for neonates with NOWS that provide a better environment for rooming-in. In addition to the physical capacity to offer rooming-in, health systems are encouraged to consider additional infrastructure supports to encourage mothers to make use of rooming-in, such as delivered meals and treatment support (e.g., methadone dosing; support groups).

To the authors’ knowledge, this qualitative study is one of first to describe the contextual challenges of implementing evidence-based care for NOWS treatment, the findings have limitations. First, participants were sampled from a single hospital setting and included nurses only. In addition, participants were mostly female and Caucasian. Participants may have participated in the study because they felt passionately (positively or negatively) about caring for mother and neonates affected by NOWS which may have impacted results. Consequently, it is important to consider replicating this study at other hospitals and with other types of healthcare professionals.

## Conclusions

This study provides a more comprehensive understanding of the barriers and facilitators that affect implementation of maternal involvement in nonpharmacological care of neonates with NOWS. As maternal involvement is essential to the care of neonates with NOWS results in improved outcomes, it is critical to recognize and understand how the clinical and community context can help or impede implementation of evidence-based care. This study identified important contextual factors relevant for NOWS care including the importance of education for staff and mothers, the role of interdisciplinary and intradisciplinary communication across the continuum of care, the need for flexible nurse staffing models, design of pathways of care pre and post discharge, and considerations regarding how the unit architecture and influence outcomes. Future implementation efforts, especially those interested in implementing nonpharmacologic approaches (e.g., Eat, Sleep, and Console), must consider the context factors and individual factors affecting implementation.

## Supplementary Information

**Additional file 1.**

## Data Availability

The datasets used and/or analyzed during the current study are available from the corresponding author on reasonable request.

## Abbreviations

NOWS: Neonatal opioid withdrawal syndrome
NAS: Neonatal abstinence syndrome
OUD: Opioid use disorder
NICU: Neonatal intensive care unit
CFIR: Consolidated framework for implementation research
FBC: Family birth center
