# Supplementary material for: Elucidating the context for implementing nonpharmacologic care for neonatal opioid withdrawal syndrome: a qualitative study of perinatal nurses
Source: BMC Pediatr. 2021 Nov 4;21:489. doi: 10.1186/s12887-021-02955-y (PMC8567648; doi:10.1186/s12887-021-02955-y)
Supplement: Supplementary file 1 — Additional file 1. [file 12887_2021_2955_MOESM1_ESM.docx]

Structured Interview Guide

INTRODUCTION: Hello ##, my name is ##. Thank you for your participation in this study. We really value your time and appreciate your participation. We anticipate this interview will take around 45 minutes but will not go over an hour. As a reminder, this interview will be audio recorded so we don’t miss anything you have to say. Is it okay if I start the recording now?

Okay, I am now recording. *{Start recording.}*

INFORMED CONSENT: Have you had time to review the informed consent form which was presented when you enrolled on the web-based survey, you also could have downloaded it*. {If no, interviewed must review/read IC to participant.*}

Do you have any questions for me about your participation in this study?

Do you consent to participate in this interview?

Do you have any other questions before I start the interview?

INTRO SCRIPT: For this interview, we are interested in your thoughts and experiences as a perinatal nurse regarding couplet care, especially for mothers who used substances during pregnancy, such as opioids.

| **Aim** | **Question** | **Probes** |
| --- | --- | --- |
| 1. Describe perinatal nurse perceptions of engaging substance-using mothers in neonatal care in (1) labor and delivery units; (2) postpartum or mother-baby units; and (3) neonatal or special care units. | 1. To begin, what type of unit do you currently work on? 2. About how long have you worked on this unit? About how long have you worked in this clinical area? 3. What comes to mind when you hear “couplet care” or “maternal-infant dyadic care?” 4. Part of couplet care involves engagement of mothers. Please tell me about how you engage mothers to participate in couplet care. 5. Tell me about your experiences with providing couplet care for substance using mothers and their babies. | Couplet care is an evidence-based practice in which mothers and newborns stay together throughout their hospital stay. Care is family-centered and prioritizes their relationship.  For example, how do you encourage a mother to do skin-to-skin with her baby? Or breastfeed? Or kangaroo? Diaper changes? Attentiveness to baby’s cues?  That’s interesting. Can you tell more about that? |
| Providing couplet care in the (Labor and Delivery, Postpartum, or NIC) unit can be challenging. We are interested in your perceptions about what helps you provide couplet care for mothers with a substance-use history and their babies. | | |
| 1. Describe perinatal nurse perceptions of facilitators and barriers to promoting maternal involvement of substance-using mothers in neonatal care in (1) labor and delivery units; (2) postpartum or mother-baby units; and (3) neonatal or special care units. | 1. Can you tell me a story about caring for a NAS couplet that impacted you the most, or that you remember the most? 2. Please describe for me some of the barriers that hinder substance using mother’s involvement in infant care? 3. What, if any, barriers have mothers brought up to you in conversation about caring for their baby? 4. What, if any, barriers have coworkers or social workers mentioned to you as barriers to helping these mother care for their baby? 5. What, if any, are the organizational or unit barriers to couplet care for these women? 6. Now can you describe some of the facilitators that help substance using mothers become more involved in infant care? 7. What are some existing strategies or perhaps new strategies that would help mothers more actively engage in couplet care with their baby. 8. What are some strategies that may help nurses and other clinicians engage substance-using moms in couplet care? | Please tell me more about that.  For example, some organizations may not have a policy or recommendation about providing care for NAS couplets specifically?  Facilitators may include things your unit/organization does, things done in prenatal care, etc.  What do moms need to actively engage with their new infants?  In other words, what types of resources and supports are needed? |
| Care of mothers and infants exposed to opioids prenatally often occurs on more than one unit. For example, mothers deliver in L&D, transition to mother-baby, and then baby is admitted to NICU for treatment of NAS symptoms. Providing coordinate and congruent care is very important but can be challenging when numerous units are involved. | | |
| 1. Describe perinatal nurse perceptions of intra- and interunit barriers and facilitators to promoting and coordinating efforts for congruent engagement of substance-using mothers in neonatal care from labor through discharge. | 1. Tell me about the path or trajectory of substance-exposed couplets from admission to your unit through discharge from your unit. 2. What are facilitators currently in place to assist in congruence of care for mother and baby in your own unit, such as, from shift to shift? 3. What, if any, are the barriers in your unit to providing coordinated, congruent couplet care for these dyads? 4. What are strategies and actions that could help address these barriers? Who is involved in these strategies and actions? 5. Now, think about their entire hospital stay and describe their trajectory as they enter the hospital for labor and are discharged later from the hospital. Tell me about the path or trajectory of substance-exposed couplets through the department or hospital from admission to discharge. 6. What are the barriers to providing coordinated/congruent care for mother and baby across hospital units? 7. What are the supports/resources that help maintain couplet care across these units? 8. When the mom and/or infant are discharged, what information or education is given to encourage maternal-infant bonding, breastfeeding, etc. following discharge from the hospital? | For example, you work in the NICU, a newborn was just admitted to your unit. Please talk to me about their time on your unit, specifically, regarding involvement with the mother.  That’s interesting, tell me more about…  Talk to me about what is communicated between nurses during transitions from one unit to another.  Think about the pregnant mom arriving in LD for delivery. Now tell me about their trajectory together until they are both discharged. Pay particular attention to their time together as a couplet and time apart.  What information or resources would be helpful? |

DEMOGRAPHIC: We have a couple more remainder questions just to learn a little bit more about who you are. These are demographic questions, and you can choose if you would like to answer them or not. If you do not want to answer, simply say “I prefer not to answer.”

Approximately how many years have you been a registered nurse?

Approximately how many years have you worked at St. Cloud Hospital?

Approximately how many years have you worked on your current unit?

What is your age in years?

How would you describe your sex (female; male)?

How would you describe your race or ethnicity (Caucasian, African-American, American Indian or Alaskan Native, Pacific Islander, Asian, more than 1, other)?

Are there any other comments you would like to make about neonatal abstinence syndrome or maternal substance abuse?

Okay, I have turned off the recording. *{Turn off recording.}* Lastly, in order to give you your $20 cash gift card I need a little bit of information. This information will only be used to distribute your gift card. Could you please tell me the name and mailing address you would like the gift card to be sent to?

CLOSING SCRIPT: Those are all of the questions I have for you today. Thank you again for your participation in this study. Have a nice day.
